# Gene expression model (in)validation by Fourier analysis

**DOI:** 10.1186/1752-0509-4-123

**Published:** 2010-09-03

**Authors:** Tomasz Konopka, Marianne Rooman

**Affiliations:** 1 BioSystems, BioModeling and BioProcesses Group, Université Libre de Bruxelles, CP165/61 Brussels, Belgium

## Abstract

**Background:**

The determination of the right model structure describing a gene regulation network and the identification of its parameters are major goals in systems biology. The task is often hampered by the lack of relevant experimental data with sufficiently low noise level, but the subset of genes whose concentration levels exhibit an oscillatory behavior in time can readily be analyzed on the basis of their Fourier spectrum, known to turn complex signals into few relatively noise-free parameters. Such genes therefore offer opportunities of understanding gene regulation quantitatively.

**Results:**

Fourier analysis is applied to data on gene expression levels in mouse liver cells that oscillate according to the circadian rhythm. Several model structures in the form of linear and nonlinear differential equations are matched to the data and it is shown that although the considered models can reproduce many features of the oscillatory patterns, some can be excluded on the basis of Fourier analysis without appeal to prior knowledge of regulatory pathways. A systematic method for testing models is also proposed based on measuring the effects of variations in gene copy-number on the expression levels of coupled genes.

**Conclusions:**

Fourier analysis is a technique that is well-adapted to the study of biological oscillators and can be used instead or in addition to conventional modeling techniques. Its usefulness will increase as more high-resolution data become available.

## Background

Transcriptional regulation of gene expression determines the way concentrations of gene products (RNA and proteins) change in time within a cell. Its study is relevant to the understanding of all biological systems and thus deserves to be treated in the most detailed manner possible. This involves modeling gene regulation mathematically.

**Figure 1:**
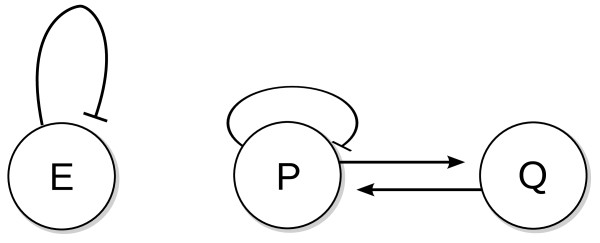
**Two genetic regulation networks**. Two genetic regulation networks capable of producing oscillations. Circles denote genes or gene products. Arrows and lines with bars denote activating and repressing actions, respectively.

Within the currently accepted paradigm, genetic processes are described as chains of interactions between segments of DNA (basically promotor sequences), RNA and/or proteins, sometimes mediated by other molecules. The understanding of a particular process at the most basic level thus involves identifying those substances, among thousands present in a cell, that take part in the interactions. This can be done experimentally although procedures can be time-consuming if, as is often the case, several substances take part in a reaction or pathway. The end result is a network of relationships which can often be summarized in terms of gene regulation diagrams such as those shown in Figure 1. Those diagrams can communicate whether or not one substance has an effect on another and if it does, whether the effect is activating or repressing.

Rough models based on regulation diagrams can be very useful to build intuition about the relation between network topology and function in static [1] as well as dynamic contexts [2,3]. In particular, they have shed light on how types of regulatory patterns such as positive and negative feedback loops exerted by proteins on the expression of their own or other genes can give rise to sustained oscillatory expression patterns associated, for example, with circadian rhythms that allow adaptation of an organism to environmental variations caused by the succession of days and nights [4]. In this context of cellular oscillators, the rough models can help verify whether proposed complex pathways can indeed give rise to stable oscillations [4] or aid in the construction of artificial systems [5,6].

Their uses notwithstanding, gene regulation diagrams also have some important limitations. One of these is that they are inherently simplified descriptions. As such, mathematical models based on regulation diagrams can provide qualitative information but should be rigorously checked against data before they can be said to capture all the relevant aspects of a genetic process. Another limitation is that the diagrams do not contain sufficient information to recreate the details of the dynamics of the interactions they illustrate. The diagrams do not explicitly encode information about the time dependence of interactions and therefore cannot be used to unambiguously write the correct mathematical model associated with the genetic process they are meant to describe. As a result, many inequivalent mathematical models can be formulated based on a single diagram. Indeed, several equation structures have already been developed to describe experimentally observed oscillatory signals, e.g. [2-4,7,8]. Not only are the parameters of the equations representing such models left unspecified, but the form of the equations themselves also shows considerable variability.

To claim a quantitative understanding of a genetic process, it is desirable to determine the associated equations with considerable confidence [9]. This implies that the form of the equations used should be validated and all the parameters in the equations should be fixed by comparison with experimental data. Once backed by such analysis, a genetic regulation model should be able to predict with confidence the behavior of a system under novel conditions. It would also provide a firm foundation for coupling a biological oscillator, for example of the circadian oscillator, to the rest of the gene regulation networks which it may be expected to influence to various degrees. However, to date, this kind of detailed understanding has not been achieved for any genetic process.

Given that quantitative knowledge about genetic processes is desirable, the question arises as to how to acquire it. Part of the difficulty lies in the fact that obtaining reliable data (i.e. relatively free of measurement and sample noise) about a single genetic process is not straightforward. This is due to a combination of factors, among which are the relative young age of some experimental techniques (such as micro-arrays) and the intrinsic stochastic nature of intra-cellular processes. The problems with noise can be overcome in two ways. First, the measurement techniques may be refined to reduce the noise. Second, experimental as well as theoretical efforts can be focused on those studies which can lead to quantitative knowledge despite of the noise. Both approaches can be pursued, but the second one is particularly interesting because it allows to make progress using technology that is already available.

Oscillatory signals are ideal candidates for precision studies of any process. Because oscillations are repetitive, it is, in principle, possible to collect data over long periods and thus obtain enough statistics to measure multiple properties of a signal even in the presence of background noise. As already mentioned, some genes have been observed to oscillate at the circadian rhythm [4]. Others are known to be periodically expressed within the cell cycle [10]. Experimentally, they can be studied using fluorescence techniques (e.g. [5,6,11]) as well as micro-array approaches (e.g. [10,12-16]). The focus of this work is to show that such data, often collected at the systems-scale, can encode qualitative as well as quantitative information about regulatory mechanisms and to describe a methodology by which this information can be extracted. The cornerstone of the presented approach is Fourier analysis, a classic technique which provides a fairly noise-insensitive way to parameterize the shape of an oscillatory signal in terms of the strengths of its harmonic components. It is shown that this technique can be used to discriminate between regulation mechanisms, and in so doing provide the opportunity to validate and/or invalidate candidate models. The methodology is suited to the bottom-up approach to modeling gene regulation networks, where the complete network is divided into loosely connected subnetworks, each containing only a few genes.

The results presented below are divided into two main parts. The first part introduces a linear model of gene regulation based on the simple harmonic oscillator. This model is exactly solvable and is thus a good starting point for a discussion of oscillating gene expression. It provides a simple framework in which to discuss oscillations and how their properties depend on model parameters. The second part extends the ideas presented in the simple harmonic oscillator context to nonlinear oscillator models and introduces modeling by Fourier analysis. The discussion draws on experimental data on circadian oscillations [12] collected using micro-array technology. The modeling techniques are applied to identify the possibilities, and sometimes lack thereof, for selecting between two oscillator models.

## Results and Discussion

### Positive simple harmonic oscillator

The most well-known oscillatory functions are sin (*ωt*) and cos (*ωt*), where *ω *is a real number representing the frequency of oscillation. They satisfy the simple harmonic oscillator (SHO) equation

(1) $$\frac{{\text{d}}^{2}s(t)}{\text{d}t^{2}}=-\omega^{2}s(t).$$

The equation can be equivalently written in terms of two first order equations

(2a) $$\frac{\text{d}s(t)}{\text{d}t}=r(t),$$

(2b) $$\frac{\text{d}r(t)}{\text{d}t}=-\omega^{2}s(t).$$

Here, the first equation is a definition of the function *r*(*t*), and the second equation is a reformulation of (1) in terms of *r*(*t*). The solutions to this set of equations are

(3a) $$s(t)=A_{1}\cos\left(\omega t\right)+A_{2}\sin\left(\omega t\right),$$

(3b) $$r(t)=\omega \left(A_{2}\cos\left(\omega t\right)-A_{1}\sin\left(\omega t\right)\right),$$

where the *A*_1_, and *A*_2 _are two real coefficients, which can be fixed given two initial conditions, for example values of the functions *s*(*t*) and *r*(*t*) at an instance in time.

In biological oscillators the quantities that change periodically are often concentrations. Since they are non-negative, they cannot be described directly by the functions *s*(*t*) and *r*(*t*) which are as often negative as they are positive. But it is possible to generalize the SHO equations slightly so that the solutions are offset from zero. Such equations are

(4a) $$\frac{\text{d}S(t)}{\text{d}t}=-C_{S}+\alpha^{2}R(t),$$

(4b) $$\frac{\text{d}R(t)}{\text{d}t}=+C_{R}-\beta^{2}S(t).$$

*R*(*t*) and *S*(*t*) are here new functions and *α*, *β*, *C_S_*, and *C_R _*are constants. The solutions are now

(5a) $$S(t)=\frac{C_{R}}{\beta^{2}}+A_{1}\cos\left(\omega t\right)+A_{2}\sin\left(\omega t\right),$$

(5b) $$R(t)=\frac{C_{S}}{\alpha^{2}}+\frac{\beta }{\alpha }\left(A_{2}\cos\left(\omega t\right)-A_{1}\sin\left(\omega t\right)\right),$$

with *ω *= *αβ *and *A*_1_, *A*_2 _real constants analogous to those in Eq. 3. Generalizations of this model are discussed in Additional file 1.

From the mathematical standpoint, all the constants in Eq. 4 and Eq. 5 are arbitrary but for biological applications, they should be chosen appropriately so that the functions *S*(*t*) and *R*(*t*) are non-negative for all values of *t*. Under such restrictions, the system can be said to describe a Positive Simple Harmonic Oscillator (PSHO) and can be applied to the biological context to describe two substances, RNA or protein, whose concentrations oscillate with the same frequency.

As to the biological interpretation of the equations, Eq. 4a suggests that substance *S *is produced at a rate proportional to the concentration of substance *R*, with the constant of proportionality equal to *α*^2^. Thus *R *can be regarded as an activating transcription factor for *S*. Substance *S *is removed at a constant rate *C_S_*. Within a cell, if *S *represents a protein concentration, this may occur if the removal is carried out by an active transport system functioning at full capacity or if it is degraded by other molecules. If *S *represents an RNA concentration, this may describe translation into protein.

The other equation for the PSHO system, Eq. 4b, suggests substance *R *is produced at a constant rate *C_R _*independently of *S*. Substance *R*, however, is removed from the system at a rate proportional to *S *with proportionality constant *β*^2^. This may occur if *S *takes part in the active degradation of *R*. Arguably, it may also be interpreted as a particular repressor mechanism.

Since the solutions of the PSHO system can be written analytically, it is possible to understand precisely how each biological process affects the temporal expression profile for the two substances. In particular, the solutions can be used to make predictions that may otherwise seem non-obvious or even counter-intuitive. For example, the period of oscillation for both quantities does not depend on either of the constant rates *C_R _*and *C_S_*, the long term average concentration of a substance does not depend on the production rate or degradation rate associated with that substance (it only depends on the rates of the other substance), and the ratio of amplitudes of oscillation of the two substances is *α/β*.

#### Fitting to data

Given experimentally measured signals for two substances whose concentrations oscillate, it may be possible to infer numerical values for the unknown parameters and constants of the PSHO model. In this section, it will be assumed that the measured signals are clear enough so that two signal amplitudes, two signal frequencies, two offsets or long-term average levels, and one phase difference between the signals can be estimated with reasonable accuracy. These are seven independent quantities.

Since the PSHO model predicts unambiguously that *R *and *S *oscillate at the same frequency and with a set phase difference, the experimental signal should exhibit these properties within a reasonable accuracy. If the signals do oscillate at the same frequency, then the six remaining measured quantities fix the six unknown constants in Eq. 5. If phase information is in fact lacking, there may remain a two-fold ambiguity due to the impossibility to identify which experimentally measured signal corresponds to each variable in the model.

Additional information extracted from the measured data can be used to validate or reject the model. In particular, since the model predicts oscillatory signals of a single frequency, measurement of more complex waveforms would be sufficient to reject the model. Such invalidation would be indicative of different interactions between the substances than those captured by the linear equations.

#### Gene-knockout and copy-number mutants

The mathematical model and its solution can also be useful when studying mutant organisms. Two types of mutants are particularly interesting: gene-knockout and copy-number mutants.

If one of the genes becomes dysfunctional, i.e. it is knocked-out, then the expression level of that substance should be expected to be nill. Assuming that the knocked-out gene is *R *and inserting *R*(*t*) = 0 into the equation for *S*(*t*) gives d*S*(*t*)/d*t *= -*C_S_*. The solution to this equation is not positive at all times and thereby indicates a breakdown of the model. Still, since the nonzero term in the above equation has a negative effect on *S*, the model may be argued to suggest a lower concentration of *S *in the knockout mutant than in the wildtype.

If the knocked out gene is *S *(*S*(*t*) = 0 at all times), the PSHO equations reduce to d*R*(*t*)/d*t *= +*C_R_*. The solution is unbounded from above and therefore also signals a breakdown of the model. However, it may still be argued that the concentration of *R *are likely to be higher in the mutant than in the wildtype.

In both cases, knocking out a gene changes the system dramatically. The equations can be used to give a general idea of what to expect in the mutant data, but the PSHO model is incapable of describing in detail the dynamics within the mutant organism.

Another type of mutant organism, a copy-number mutant, contains not one but two copies of a particular gene and associated promotor sequence. Supposing this gene is *R *and assuming that the production rate of *R *in Eq. 4b is proportional to the probability for a transcriptase to encounter *R*'s promotor site, it is reasonable to assume that *C_R _*should increase twofold as a result of the gene replication leaving the other parameters in the model unaltered [17,18]. The change in *C_R _*has two distinct effects on the analytic solutions of the model. It alters the coefficients *A*_1 _and *A*_2 _and thus the amplitude of both the *R *and *S *expression signals, albeit in a manner that depends on the initial conditions. Doubling *C_R _*also changes the offset of the *S *signal by a factor of two and this is an unambiguous and verifiable effect.

If the replicated gene is *S*, it is the production rate of *S *parameterized by *α*^2 ^that is likely to double leaving other parameters unchanged. *α *appears in several places in the analytical solution and is thus responsible for more than one effect in the copy-number mutant: the average level of gene product *R *should decrease by a factor 2; the ratio of amplitudes for *S *and *R *should decrease by $\sqrt{2}$; the frequency of oscillation should increase by a factor $\sqrt{2}$. All three signatures are discrete and unambiguous. The amplitudes of each substance may also shift but the extent of the change will depend on the initial conditions.

If experimental data on the knockout and copy-number mutants were available, their signals could be compared with these predictions and thus used to validate the model or to identify its weaknesses. The two types of mutants would test the model in different ways, with the copy-number mutants arguably yielding more subtle and valuable information.

### Nonlinear oscillators

Most interacting systems other than the simplest ones such as the PSHO model in Eq. 4, are described by nonlinear equations. This presents several challenges.

First, there are an infinite number of mathematically distinct non-linear models that can give rise to oscillations. Such models, for example, can be based on differential equations or perhaps include stochastic components. In the biological context, it may sometimes be possible to restrict attention to certain types of models on the grounds that they may be based on previous knowledge of interactions or on commonly used assumptions about kinetics of chemical reactions (e.g. with terms of the Michaeles-Menten type). However, it is important to emphasize the assumptions of such models.

Second, even if attention is focused on a small set of nonlinear models, analytic solutions describing the oscillations they produce in general will not be available. Thus, the only way to proceed is by numerical methods.

Third, whereas all the parameters of the PSHO model can be estimated using only coarse features of a measured expression level signal - period, amplitude, and offset, - the same may not hold for many nonlinear models because these generally have many more unknown parameters. The situation may be alleviated if some of the model parameters can be given values using data from complementary experiments. But unless this can be done, the fitting procedure based only on period, amplitude, and offset will leave many parameters undertermined.

#### Fourier spectrum

One way to tackle the last challenge and make progress in determining model parameters is by analyzing the finer features of the oscillating signal. The key observation is that oscillating signals produced by a nonlinear model cannot be ideal sinusoids. Instead, they can be decomposed into a sum of sine and cosine functions of different frequencies.

From a mathematical standpoint, an oscillating function *H*(*t*) can be written as

(6) $$H(t)=c_{0}+\sum_{p=1}^{\infty }c_{p}\sin\left(p\omega t\right)+d_{p}\cos\left(p\omega t\right)$$

with *ω *a base frequency and *c*_0_, *c_p _*and *d_p _*some real coefficients. These coefficients completely determine the function, meaning that it possible to reproduce the waveform by listing the coefficients. In effect, therefore, the waveform can be thought of as a function of *p*, i.e. *H*(*p*) (see [19,20] and Additional file 1 for more details). When applied to Eq. 6, its magnitude is *H*(0) = *c*_0 _and $H(p)=\sqrt{c_{p}^{2}+d_{p}^{2}}$. It is called the Fourier transform, or the spectrum, of *H*(*t*) and can also be computed using a well defined formula (or computer packages) when the waveform is given as a sequence of values at regularly spaced intervals. The spectral representation of an oscillating signal, widely used in several branches of science and engineering, is useful for several reasons. First, it transforms a continuous signal (or a time-series of measurements) into a short list of coefficients. This implies that the shape of the oscillating waveform can be described accurately and quantitatively in terms of only a few numbers. Second, since an entire set of observations is used to compute every coefficient, the uncertainty associated with that coefficient may be small even if the uncertainty on each individual data point in the time series is fairly large. Furthermore, accuracy increases with greater number of observations per period and also with a greater number of observed periods. Properties of Fourier spectra are further discussed in Additional file 1.

### Application to circadian oscillations

The Fourier transform can be applied to data from any biological oscillator. For concreteness, however, this section focuses on oscillations in gene expression due to the circadian cycle, data for which was recorded in a time-series micro-array experiment on mouse liver cells [12]. The experiment was performed at high temporal resolution, recording 48 points in one-hour intervals over two complete periods of the circadian cycle and identifying more than 3,000 transcripts that exhibit oscillatory behavior. Some of the transcripts have previously been known to oscillate and have been included in qualitative modeling efforts (e.g. [4]). Many others, however, are not part of most circadian oscillation models, and therefore their coupling with the circadian cycle and other external factors are not known. Both types are interesting for detailed modeling.

**Figure 2:**
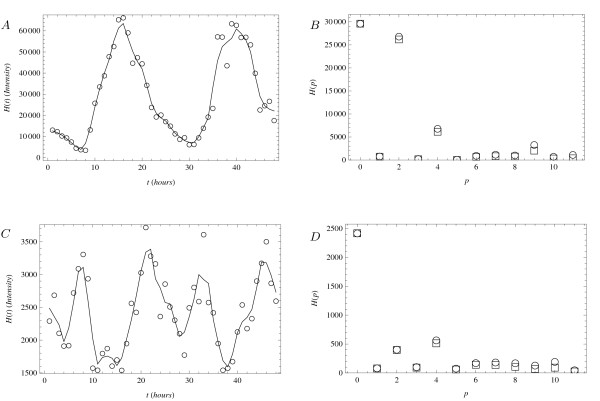
**Gene expression in mouse liver cells**. Expression levels of genes in mouse liver cells [12]. (Top): gene Nr1d2 (probeset 1416958_-_at). (Bottom): gene Pde12 (probeset 1454963_-_at). (Left column): raw data points (circles) and a smoothing curve (line) computed using a three-hour simple moving average. (Right column): spectra for the unsmoothed (circles) and smoothed (squares) data. The spectrum component at *H*(*p *= 1) corresponds to *ω *= 2*π*/48 hours^-1^.

**Figure 3:**
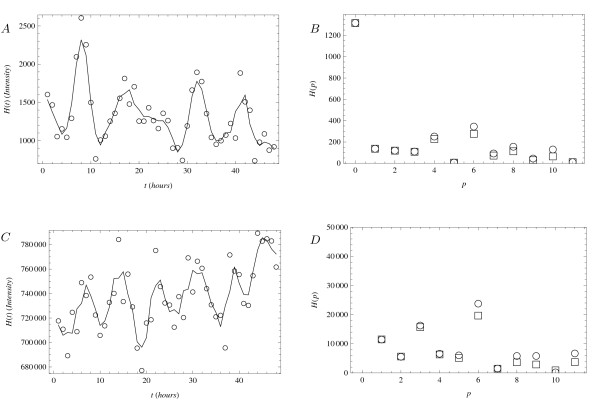
**Gene expression in mouse liver cells**. Expression levels of genes in mouse liver cells [12]. (Top): gene Eif2ak3 (probeset 1449278_-_at). (Bottom): gene Ttr (probeset 1455913_-_x_-_at). (Left column): raw data points (circles) and a smoothing curve (line) computed using a three-hour simple moving average. (Right column): spectra for the unsmoothed (circles) and smoothed (squares) data. The spectrum component at *H*(*p *= 1) corresponds to *ω *= 2*π*/48 hours^-1^.

Figures 2 and 3 show a small sample of the data on circadian oscillations in mouse liver cells [12] for the purposes of the present discussion. In the left column, the plots show the expression levels and, in the right column, they show the corresponding Fourier spectra. The genes in Figure 2 are Nr1d2 (top row), member of a nuclear receptor family, and gene Pde12 (bottom row), coding for an enzyme. The genes in Figure 3 are Eif2ak3 (top row), a translation initiator factor, and Ttr (bottom row), a serum carrier. Among these, only the first gene (Nr1d2) has been previously noted to participate in the regulation of the circadian rhythm [12].

In the top row of Figure 2, the peak at *p *= 0 in the spectrum corresponds to the offset of the expression level from zero, and the peak at *p *= 2 confirms the frequency of oscillation is such that two complete cycles are completed during the observation period. Indeed, there are only two clear maxima and minima in the top-left panel indicating a circadian cycle with a period of 24 hours. The *p *= 2 peak is large relative to the noise (the values of the spectrum *H*(*p*) at large *p*) so the experimental detection of oscillations for the gene can be said to have been made with high confidence [12]. The fact that the heights of *p *= 2 peaks computed from the smoothed and unsmoothed data are similar further strengthens this conclusion.

In addition to the *p *= 0 and *p *= 2 peaks, the spectrum also shows a peak at *p *= 4. This is also above the noise and reveals by how much the shape of the observed oscillating pattern differs from a pure sinusoid. The other elements of the spectrum are near zero, indicating that they are either not present or that it is difficult to distinguish them from the background noise.

In the bottom row of Figure 2, the peak at *p *= 0 again corresponds to the offset of that gene's expression from zero. Other clear peaks appear at *p *= 2 and *p *= 4. The existence of the peak at *p *= 2 indicates that the base frequency of that oscillating signal is the same as in the top panel. In other words, in the strict sense the period of the second oscillating signal is the same as that of the first - 24 hours. The spectrum, however, shows a pronounced component at *p *= 4 and because it is in fact larger than the one at *p *= 2, the bottom-left panel actually shows four main maxima and minima. This double-peak signature, evident only in the Fourier spectrum and not in the original representation, will lead to an interesting result in the next section.

Figure 3 shows two other sets of signals and spectra. Again, peaks at *p *= 0 correspond to offsets from zero (in Figure 3D this peak is not visible because the vertical scale is optimized to show the spectrum features at higher *p*). The structure of the other peaks shows some important differences from the previously discussed spectra. In panels Figure 3A and Figure 3C, the expression levels exhibit a trend - downward in the first and upward in the latter. In the frequency domain representations in Figure 3B and Figure 3D, this translates into nonzero levels at several values of *p *[19,20]. In particular, the spectra at *p *= 1 are larger than the noise level and larger than in Figure 2 because of this effect. The first indications of oscillations appear at *p *= 4 in Figure 3B and at *p *= 3 in Figure 3D, suggesting that their base periods are 12 and 16 hours respectively. In both cases, however, the strongest component appears at *p *= 6, which corresponds to an oscillatory component with period of 8 hours. These combinations of features will also lead to concrete results in the next section.

#### Model selection

Many gene regulation network diagrams and associated equations can lead to oscillatory behavior. Model selection is then the task of determining if a particular model structure is a good description of certain experimental data. To demonstrate the role Fourier analysis can play in this processes, two specific model structures can be considered. Their diagrams are shown in Figure 1. (A simpler example of model selection using Fourier spectra is discussed in Additional file 1.)

The first model describes a single substance *E *that inhibits its own production after a time delay. The equation for the model,

(7) $$\frac{\text{d}E(t)}{\text{d}t}=\frac{\alpha_{E}}{1+{\left(E(t-\delta_{A})/\beta_{E}\right)}^{n_{E}}}-k_{E}E(t),$$

has previously appeared in the literature as a toy model for circadian oscillations [7]. It is discussed here because, despite containing five unknown parameters (*k_E_*, *α_E_*, *δ_E_*, *β_E_*, and *n_E_*), it is relatively simple.

The second model involves two substances *P *and *Q*. Their dynamics is given by

(8a) $$\frac{\text{d}P(t)}{\text{d}t}=\frac{V_{P}X_{PQ}}{W_{P}+X_{PQ}}-k_{P}P(t)$$

(8b) $$\frac{\text{d}Q(t)}{\text{d}t}=\frac{V_{Q}W_{Q}}{W_{Q}+X_{PQ}}-k_{Q}Q(t)$$

where

(9) $$X_{PQ}=\left[P(t-\delta_{P})-Q(t-\delta_{Q})\right]\Theta \left(P(t-\delta_{P})-Q(t-\delta_{Q})\right)$$

involves the Heaviside function Θ (Θ(*z *≥ 0) = 1, Θ(*z *< 0) = 0). This model also appeared previously as a toy model for circadian oscillations [8]. It has several more parameters (*V_P _*, *V_Q_*, δ*_P_*,δ*_Q_*, *k_P_*, *k_Q_*, *W_P _*and *W_Q_*) than the first model, but it is discussed here because its equations describe a regulatory mechanism that uses a nontrivial logic operation encoded by the Heaviside function.

#### Fitting spectral signatures

The models in Eq. 7 and Eq. 8 can be solved numerically for any choice of parameters and the signal thus produced analyzed in terms of its Fourier spectrum. These spectra, to repeat from the previous section, contain all information about the signals. The coarse features of the signal are encoded in the two leftmost peaks: the signal offset in the height of the leftmost peak; the oscillation amplitude in the height of the next peak; the oscillation frequency in the distance between adjacent peaks. The heights of the subleading peaks in the spectrum are independent quantities whose values derive from the shape of the oscillating waveform. As shown in Figure 2, spectra derived from recent experimental data [12] can yield numerical values for four independent quantities: the three coarse features of an oscillating signal plus the first subleading feature (the remaining subleading features being confused by noise). The fact that the data shows a nonzero subleading component is sufficient to immediately invalidate the Positive Simple Harmonic Oscillator model. The biological scenario described in the context of that model can therefore be ruled out in the context of the circadian oscillations. The other models, Eq. 7 and Eq. 8, however, are viable.

The number of measured independent quantities is one fewer than the number of parameters in the single-gene model of Eq. 7. Naive parameter counting therefore suggests that the model should be able to fit the experimental data. The two-gene model of Eq. 8 contains even more parameters and so it too should be able to fit the data. Indeed, parameters for both models can be found with relative ease that give rise to spectra that resemble Figure 2B. Therefore, additional information must be input before one can be selected over the other. This information can be in the form of restrictions on the values the parameters can take in each model, for example derived from independent experiments, or from precise measurements of further features in the oscillating gene's spectrum. (Arguably, the additional information may also come in subjective form. For example, in this situation Occam's razor marks the model with a single gene as preferable over the one with two genes.)

The situation is different when matching the models with the spectrum in Figure 2D, which has the unusual feature that the higher frequency component is stronger than the lower frequency one. Assuming that this signature is not an artifact of noise, the two models can be tested for their ability to reproduce such a spectrum by generating sets of parameters, Fourier-transforming the models' output for each parameter set, and comparing the strengths of the relevant peaks. Since the volume of the parameter space for each model is large, this cannot be done exhaustively. But it can be done by sampling a region of the parameter space of interest. For this study, the region of interest can be defined as ranging from 0:01 to 100 for most parameters in the two models (the parameter *n_E _*was taken as equal to 2, 3 and 4).

When sampling the parameter region of interest uniformly at random on a logarithmic scale for each model, the result, after thousands of samples, is that no parameter set of the single-gene model of Eq. 7 gives the desired Fourier signature (data not shown). The two-gene model of Eq. 8, in contrast, does yield a few sets of parameters for which the peaks in the spectrum have relative strengths as in Figure 2D. Accepting the results of random sampling study implies the rejection of the single-gene model for describing the behavior shown in Figure 2D. Since the model is only a toy model and does not describe any interactions between chemicals in a cell, it may not be totally surprising that it is inadequate to fully capture the intricacies in the data. But the analysis is nonetheless an example of how Fourier analysis may help in model selection in gene regulation science. In contrast, if model fitting were to be done using the original oscillating waveform and associated methods (for example by minimizing the deviation between experimental and model signals), invalidation of the single-gene model may not be as clear because the imperfect overlap between the fitted model and data may be blamed on noise. (A related example is discussed in more detail in Additional file 1.)

Similar model invalidation arguments can be directly applied to all genes in the mouse liver cell data set [12] with features similar to those in Figure 2. In some cases, they can be extended to other genes with more peculiar features as well. For example, both models in Eq. 7 and Eq. 8 can be argued to not describe the data in Figure 3 very well because they do not produce signals with long-term downward/upward trends. Furthermore, even if such trends are ignored, comparing the peak structures in Figure 3 and in Figure 2 can lead to interesting conclusions. In Figure 3B, the two detectable peaks are not at frequencies related by an integer multiple. Since neither the single-gene model nor the two-gene model can produce such signals, both models must be rejected in that context. In Figure 3D, the frequencies of the two peaks are multiple integers of each other, but their base frequency (corresponding to a period of 16 hours) is not equal to the circadian rhythm. In the two-gene model of Eq. 8, substances *P *and *Q *always have the same period, so detection of the signal in Figure 3D implies that the gene cannot be a partner to another gene belonging to a class represented by the genes shown in Figure 2.

Model invalidation carries information about regulatory mechanisms for genes under the influence of the circadian cycle. For example, since the expression profile of the gene in Figure 2C is inconsistent with a self-regulatory mechanism, it suggests that the gene is strongly regulated by more than one substance. The additional regulatory factors may be in the form of other genes actively participating in the transcription process as in the second model or in the form of metabolic or other systemic cues. Indeed, the latter hypothesis was suggested and indeed tested in [12]. These arguments are indicative and not conclusive, but it is nonetheless interesting that they can be made at all on the basis of two signals from a micro-array experiment and a model selection analysis.

Equally important, the fit of the single gene model to the expression profile of the gene in Figure 2A suggests that gene is less affected by the same external conditions. That is, it is more weakly coupled and hence more robust to external perturbations.

#### Exploiting mutants

Another technique useful for model selection exploits mutant oscillators, particularly copy-number mutants, similarly as described in the context of the positive simple harmonic oscillator. Since there is currently no experimental data similar to [12] for copy-number mutant organisms, the discussion here uses signals generated in-silico.

The task is to determine, given two oscillating signals/substances *A *and *B*, whether they are produced by a regulation scheme described by the first model applied to each substance separately, or whether their dynamics is better described by the second model that contains a particular form of coupling. The task is approached by comparing the original oscillations to those produced by mutants in which the number of coding regions for substance *A *is changed. If it is changed from one to two, the mutant can be called a copy mutant. If the copy-number is further increased to an integer *N*, the mutant can be called an *N*-copy mutant.

To accommodate this scenario using the first model, its mathematical description must be first expanded to include two independent oscillating variables. This can be done by defining a new quantity *E' *governed by an equation of the same form as Eq. 7 with a new set of parameters also distinguished from the original ones by primes. Since the substances *E *and *E' *obey mathematically identical equations, the mutations in coding regions for substance *A *can be taken to lead to changes in either equation. Supposing that *A *corresponds to *E*, the mutation would not affect any of the parameters associated with *E'*. Furthermore, by the same arguments as before, the sole effect of the replicated gene may be to increase *α_E _*by a factor of two (or *N *for an *N*-copy mutant).

Using the second model, there is some ambiguity at the start since it is not clear whether the replicated gene *A *should be identified with quantity *P *or *Q*. The two possibilities must therefore be considered separately. For simplicity, however, the discussion here is focused on the case where the gene in question corresponds to *P*. The replication is therefore assumed to increase the parameter *V_P _*by a factor of two (or *N *for an *N*-copy mutant) while leaving all other parameters unchanged.

To ensure a fair comparison between the two models, they must be matched before the gene copying takes place. Ideally this should be done by matching each model to a set of experimental data for the wildtype. For the present discussion, however, it is sufficient to only match the models to each other. This can be done by first choosing a set of parameters for one model fairly arbitrarily and then adjusting the parameters of the other model to match the spectrum of the first. As an example, the parameters of the two-gene model in Eq. 8 are taken to be *V_P _*= 1.5, *V_Q _*= 0.25, *W_P _*= *W_Q _*= 0.2, *k_P _*= *k_Q _*= 0.4, *δ_P _*= *δ_Q _*= 5. Assuming that the decay constants and all delays are equal, the signal of *P *must be matched to a signal of *E *by adjusting *n*, *α_A_*, *β_A_*. By some trial and error the values *k_E _*= 0.4, *δ_E _*= 5, *n *= 3, *α*_*E *_= 1, *β*_*E *_= 0.4 are found to reproduce the spectrum peaks of *P*'s signal within 10%. The first subleading peaks turn out to be matched within about 25%.

The parameters in the models give scope to improvements in this matching procedure but this will not be important for what follows. It is important to recognize, however, that the models cannot be matched exactly, even in principle. Since there are a large number of peaks in the spectrum and only three adjustable parameters (*n*, *α_A_*, and *β_A_*), in general the matching problem will have no solutions, reflecting the fact that the two models are mathematically inequivalent.

**Figure 4:**
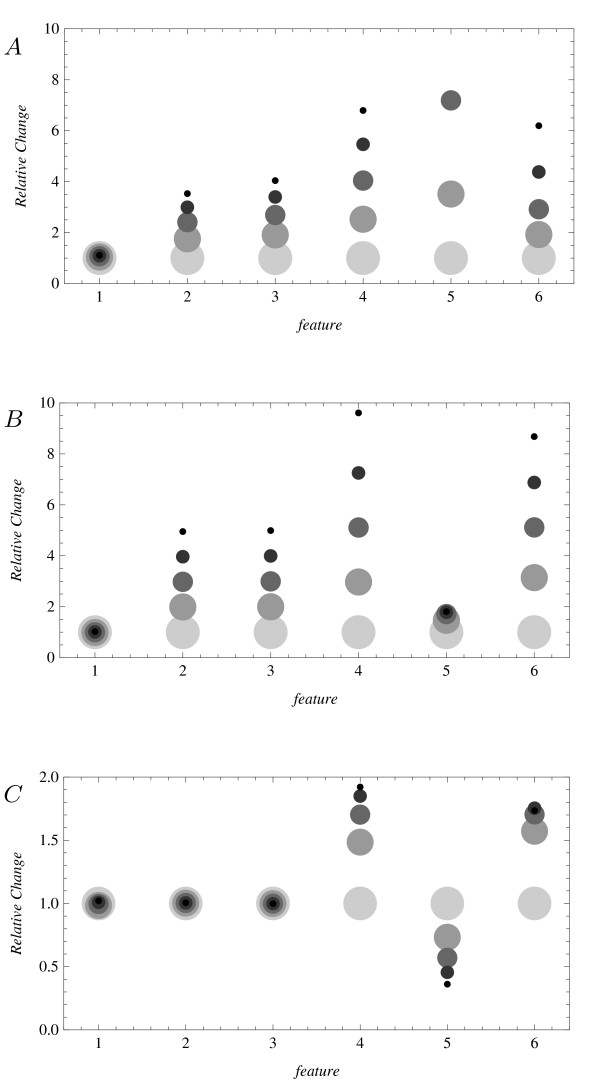
**Changes in spectra for N-copy mutants**. Effects of *N*-copy mutations in *E *and *P *on signal features in the Fourier spectrum for oscillators: *A*. *E *of Eq. 7, *B*. *P *of Eq. 8, *C*. *Q *of Eq. 8. On the horizontal axis, *f *stands for features: 1 is period, 2 is offset (analogous to *H*(*p *= 0) in Figure 2), 3 is amplitude, 4,5,6 are the first, second, and third harmonics. On the vertical axis, relative change is the ratio of feature strength in the mutant to wildtype. Marker sizes represent *N *from 1 (large) to 5 (small).

In copy-number mutants, the signals for the quantities *E*, *P *, and *Q *differ from those in the wildtype. The relative effects on various signal features (ratios of feature strengths in mutants to the original) for these quantities, using the initial parameters noted above, are shown in Figure 4. As the quantity *E' *is unaffected, it is excluded from the figure. The figure should be viewed as a representation of the types of effects that may arise when comparing models and excessive weight should not be attached to any one quantitative detail contained within it. Nonetheless, it demonstrates a few interesting points.

The figure shows that the changes in the coarse signal features can respond similarly due to mutations in the two models: the periods remain roughly at the same level for all substances and the offsets and amplitudes increase for corresponding substances - they rise for *E *and *P *and remain constant for *E' *(not shown) and *Q*. (In actuality there are small differences in the models but they are all within the initial matching accuracy of about 25%.) This observation alone is somewhat surprising. A priori, it would be reasonable to expect that even the coarse features of signal *Q *would change in response to a mutation in *P*, the first being regulated by the latter. That this need not be true indicates that the coarse features alone are not sufficient to infer causality or lack thereof. The figure also shows that the harmonic peaks (labelled by *f *= 4, 5 and 6 in the figure) can respond quite differently in the two models and that these differences can be much larger than the variations observed for the coarse features. In the figure, the difference between the responses of the second harmonic (*f *= 5) for substances *E *and *P *is particularly pronounced and could be used to discriminate between the models. Also, because the panel corresponding to *Q *shows variation in the harmonic structure after the mutation, the signature could be used to infer the existence of an interaction between *P *and *Q *even though the coarse features are consistent with lack thereof. Seen from a different perspective, the figure shows concrete predictions for gene expression profiles in copy-number mutants for organisms whose wildtype is well modeled by (7) or (8). The predictions are in the form of changes in the shape of the profile.

## Conclusions

In summary, oscillating signals can be useful resources for gene regulation analysis. Their Fourier spectra can separate gene regulation signatures from noise, fix parameters of a candidate model, test the model's predictive ability and compare it with alternatives. Importantly, these tasks can be tackled in a systematic, unambiguous, and reproducible manner. Thus, oscillatory signals provide the means, building on the qualitative understanding of gene expression and gene regulation, to begin discussing gene regulation quantitatively despite the presence of noise.

Measurements of features in Fourier spectra of oscillating genes are technologically and experimentally feasible: oscillatory gene expression has already been measured using fluorescence techniques (e.g. [5,6,11]) or inferred from time-series micro-array data (e.g. [10,12-16]). Indeed, some features of Fourier transforms have already been applied in such studies to statistically separate oscillatory from non-oscillatory genes and some studies have also focused on the difficulties associated with sampling in micro-array data [21-23]. The same technique can also be used to mine the data deeper for information about specific regulatory mechanisms.

As shown in Figure 2, the resolution and time-span of some experimental data now approaches a sufficient level to allow reliable computation of the subleading features of the Fourier spectrum. As more such data becomes available, this structure should be put to use to infer quantitative knowledge about gene expression mechanisms. In so doing, studies based on micro-array experiments can be used to infer not only systems-wide properties (such as number of oscillating genes) but also specific constraints on the dynamics of individual genes. This shows that the data collection methods developed for systems biology can be fruitful for studies familiar from traditional reductionist biology.

But fully harnessing the benefits of gene regulation modeling in Fourier space requires a dedicated approach. From the theoretical perspective, it requires studying gene regulation mechanisms that cause distinctive patterns in oscillatory gene expression signals. From the experimental perspective, it suggests there is value in measuring oscillating waveforms at higher temporal resolution, for longer times, and also under varying but controlled conditions, for example in copy-number mutants. When coordinated, the result may be the description of genetic regulation mechanisms at a level of detail that would allow the confident prediction of cell behavior under previously untested circumstances. It may also shed light on how coordination of gene expression occurs across various regulatory pathways and larger gene regulatory motifs.

## Authors' contributions

TK designed the research, performed the calculations, and wrote the paper. MR discussed the research, the calculations and the paper. Both authors read and approved the final manuscript.

## Supplementary Material

Additional file 1 **Supplementary discussion**. PDF file containing extensions of the positive simple harmonic oscillator model as well as an additional example of model selection using frequency-domain methods. Click here for file
