# Supplementary material for: Gene expression model (in)validation by Fourier analysis
Source: BMC Syst Biol. 2010 Sep 3;4:123. doi: 10.1186/1752-0509-4-123 (PMC2944138; doi:10.1186/1752-0509-4-123)
Supplement: Additional file 1 — Supplementary discussion. PDF file containing extensions of the positive simple harmonic oscillator model as well as an additional example of model selection using frequency-domain methods. [file 1752-0509-4-123-S1.PDF]

Gene expression model (in)validation by Fourier analysis - Additional file 1

Tomasz Konopka*[1] and Marianne Rooman[1]

[1] BioSystems, BioModeling and BioProcesses Group, Université Libre de Bruxelles, CP165/61 Brussels, Belgium

Email: Tomasz Konopka*- tkonopka@ulb.ac.be; Marianne Rooman - mrooman@ulb.ac.be;

*Corresponding author

Linear oscillator models

The positive simple harmonic oscillator (PSHO) defined in the main text (Eq. 4) is a system of two first-order coupled differential equations

$$\frac{\mathrm{d}S(t)}{\mathrm{d}t} = -C_S + \alpha^2 R(t), \tag{S1a}$$

$$\frac{\mathrm{d}R(t)}{\mathrm{d}t} = +C_R - \beta^2 S(t), \tag{S1b}$$

with C_S, C_R, α and β real positive constants. The solutions are the oscillatory functions

$$S(t) = \frac{C_R}{\beta^2} + A_1 \cos(\omega t) + A_2 \sin(\omega t), \tag{S2a}$$

$$R(t) = \frac{C_S}{\alpha^2} + \frac{\beta}{\alpha}\left(A_2 \cos(\omega t) - A_1 \sin(\omega t)\right), \tag{S2b}$$

where $\omega = \alpha\beta$, and A_1 and A_2 are constants describing the initial conditions of the oscillators subject to the constraint that the functions $S(t)$ and $R(t)$ are nonzero at all times.

This base model can be generalized in several ways while preserving its essential features of linearity and existence of analytic solutions. For example, additional terms can be included on the right hand side of equations (S1). For two variables, the most general linear system of equations can be written as

$$\frac{\mathrm{d}\widetilde{S}(t)}{\mathrm{d}t} = -\widetilde{C}_S + \xi_1 \widetilde{\alpha}^2 \widetilde{R}(t) - \xi_2 \widetilde{\gamma}_S^2 \widetilde{S}(t), \tag{S3a}$$

$$\frac{\mathrm{d}\widetilde{R}(t)}{\mathrm{d}t} = +\widetilde{C}_R - \xi_3 \widetilde{\beta}^2 \widetilde{S}(t) - \xi_4 \widetilde{\gamma}_R^2 \widetilde{R}(t). \tag{S3b}$$

In these equations, tildes distinguish functions and constants from those in Eq. S1. The four independent sign factors $\xi = \pm 1$ and the two self-couplings $\widetilde{\gamma}_S^2$ and $\widetilde{\gamma}_R^2$ are new parameters. Biologically relevant models have all parameters and sign factors positive, in which case the self-coupling terms produce damping. Like for the PSHO, the solutions to this general model can be written down analytically. Qualitatively, they differ from the subclass of solutions given in Eq. S2 in that their oscillations decay, effectively stabilizing the expression level after some time. This implies that matching with data is more difficult than for non-damped model.

Another form of generalization involves defining systems of equations with a larger number of functions corresponding to more complex interaction networks involving more coupled substances. Solutions to such extensions are often functions that grow in time as well as oscillate and thus their suitability as descriptions of finite biological systems must be considered on a case by case basis.

Some properties of Fourier spectra

A periodic function H can be equivalently and interchangeably represented in the time domain as $H(t)$ and in the frequency, or Fourier, domain as $H(p)$ (also see [19,20] of main text). Thus, given a function $H(t)$ on the interval $0 \le t < N$, it can be written as the expansion

$$H(t) = c_0 + \sum_{p=1}^{\infty} c_p \sin(p\omega t) + d_p \cos(p\omega t) \tag{S4}$$

with $\omega = 2\pi/N$ and c_0, c_p and d_p some real coefficients. The equality in this formula implies that the oscillatory function $H(t)$ can be equivalently described in terms of these coefficients. Such a dual description of the function, labelled $H(p)$, is called the Fourier transform of $H(t)$.

In practice, the function $H(t)$ is often given as a series of values for equally spaced values of t, for example labeled by integers $t = 0, 1, 2, \ldots, N-1$. In such cases, it is possible to compute the Fourier transform using the formula

$$H(p) = \frac{1}{N}\left[1 + \Theta(p - 1/2)\right] \sum_{t=0}^{N-1} H(t)\, e^{ip\omega t}. \tag{S5}$$

(Implementations of such transforms are implemented in most mathematical software packages such as Mathematica, Maple, or Matlab.) In this definition (including the factor involving the Heaviside function), the complex function $H(p)$ is such that its magnitudes for different p are $|H(p=0)| = c_0$ and

$|H(p)| = \sqrt{c_p^2 + d_p^2}$.

The remainder of this section reviews some properties of the Fourier transform and its use in modeling by means of an example. The discussion deals only with the magnitudes of the Fourier transform and disregards all phase information (i.e. the difference between c_p's and d_p's).

The example involves the oscillatory signal shown in figure S1A. A curve similar to that in the figure could be produced by simple gene regulation models such as those described in the main text but to avoid over-complicating the present discussion, the curve is in fact obtained using Eq. S4 with $\omega = 2\pi/60$, $c_0 = 50$, $c_4 = 45$, $c_8 = -12$, $c_{12} = 7$ and all other parameters set to zero (all units are arbitrary):

$$H_1(t) = 50 + 45\sin(4\omega t) - 12\sin(8\omega t) + 7\sin(12\omega t)\,. \tag{S6}$$

The first term on the right hand side is the offset of the signal from zero. Indeed, its value 50 corresponds to the offset observed in the figure. The second term in (S6) describes the lowest frequency component of the signal and assigns it an amplitude of 45 and a frequency of $4\omega = 2\pi/15$. These items of information can also be inferred visually from the curve in the figure since the latter has a period of 15 units and a range of not more than 50 around the average. Together, these represent what may be called the coarse features of the oscillatory signal. The subleading terms in (S6) may then be said to determine the finer features of the signal. Their existence can be inferred from the figure by the fact that the wave is not exactly sinusoidal, but they are not easily quantified without the decomposition into frequency modes.

Measurements of the signal from Eq. S6 are supposed to not yield time-intensity pairs falling on the curve, but rather some distorted values as shown by circles in the same figure. In the example, the hypothetical measurement points are selected at each time point from a normal distribution centered around the actual signal value (as determined by the curve) with a standard deviation $\sigma = 10$. Any negative values are set to zero. A set of such hypothetical measured values of the signal is shown in figure S1A. The error bars denote the standard deviation.

The aim of the example is to demonstrate signal identification and model selection in a simple setting with two candidate models. The first candidate model is the one actually responsible for producing the real signal, Eq. (S6), which gives rise to the curve in figure S1A. The second candidate is a simpler model that produces a wave given by Eq. S4 with $\omega = 2\pi/60$, $c_0 = 50$, $c_4 = 45$, $c_{12} = 7$ and all other parameters set to

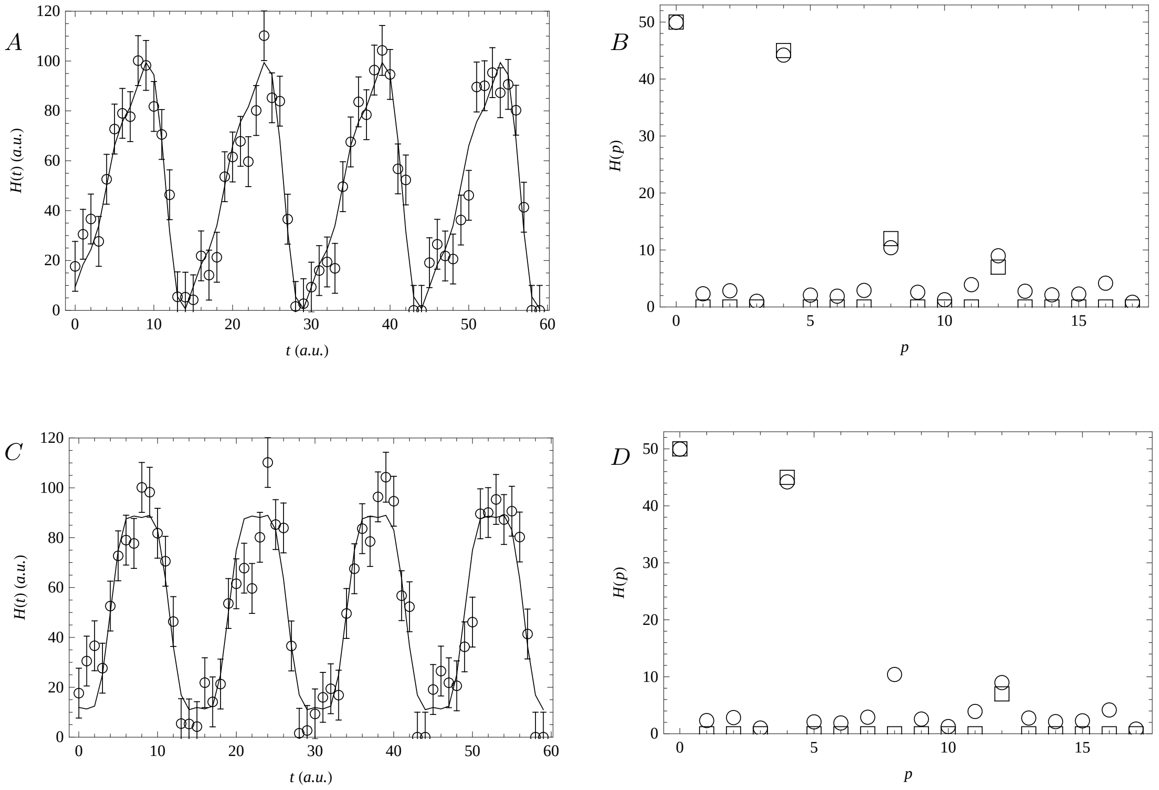

Figure S1: Fourier spectra can be used for model selection. (Left column): noisy synthetic data (points) is superimposed on two models (curves). (Right Column): Fourier spectra of the synthetic data (circles) and the models (squares). All units are arbitrary.

zero:

$$H_2(t) = 50 + 45\sin(4\omega t) + 7\sin(12\omega t)\,. \tag{S7}$$

Its waveform, superimposed on the hypothetical set of noisy measurements defined above, is shown in figure S1C. Compared with the model of Eq. S6, this contains all the appropriate terms except the one for the frequency 8ω component. Its coarse features are therefore the same as in the first model, but its finer features are not.

The task at hand is to determine which of the two models is the appropriate description of the data, or, in other words, to determine whether the simulated data is sufficiently accurate to discriminate the correct

model from the simpler decoy.

One method of analysis is to define a cost function, for example

$$D^2 = \frac{1}{N} \sum_{t=0}^{N-1} (m_t - d_t)^2, \quad \text{(S8)}$$

where m_t and d_t denote the intensity values at time t (i.e. $H(t)$) for the model and the data, respectively. The better model is the one that gives the lower cost function. In this case, the cost values are $D = 9.3$ for the first model and $D = 11.8$ for the second model, correctly identifying the first model as the better fit. However, the analysis is not entirely conclusive because both values are comparable to the standard deviation $\sigma = 10$ of the noise and it is not clear whether the simpler model that does not fit as well can be rejected/invalidated. This is because the data is noisy and it is quite acceptable for the model to miss or deviate from some data points. In the examples in the figure, the first model curve misses the shown 1σ intervals 19 times and the 2σ intervals 2 times (there are 60 points in total) The second model misses the 1σ intervals 24 times and 2σ intervals 5 times. The match of the simple model is not impressive, but becomes entirely acceptable (65% percent of points within 1σ etc.) if the 1-standard deviation interval is thought to be higher than actually shown by 20-30%, or if some clear outliers are excluded. This is not justified in the present example, but may arise in a situation where the level of noise is not known but must itself be estimated.

Another method of analysis involves computing the Fourier spectra of the oscillating signals. Portions of these spectra for low values of p are shown in Figures S1B and S1D for the first and second model, respectively. In each of the spectra, circles denote the spectrum computed from the noisy signal and the squares denote that computed from the model using an equal number of points. The spectra show a small number of peaks, which correspond to the nonzero c_p coefficients in the Fourier decomposition in Eq. S4, and a large number of values close to the axis, which are due to noise from measurement or from numerical effects in the Fourier transform algorithm.

The leftmost peaks in the spectra represent the offset values. The positions of the next peaks at $p = 4$ reveal the base frequency or period, and their heights the amplitude of that frequency component. Rough agreement of the two models with the noisy data in these parts of the spectra suggests that they both correctly capture the coarse features of the hypothetically measured oscillatory waveform. In Figure S1B, the match of the first model also extends to the other frequency components: the discrepancies in all the

peaks heights of the model and the data are comparable to the magnitude of the noise. In Figure S1D, in contrast, the peak in the data spectrum at position $p = 8$ is well above the noise level, while it is nonexistent in the model spectrum. The discrepancy is more than a matter of a few or few tens of percent (errors are discussed below), and therefore this analysis method based on the Fourier spectra shows that the model of Eq. S7 does not capture a finer feature observed in the data.

An essential difference between the time-domain and frequency-domain based methods is rooted in the number of quantities extracted from the data that are compared with the models. The first compares only one cost function while the second as many independent quantities as there are peaks in the spectrum. In the example, the first method gives an indication of a misfit between one model and the data but is not entirely conclusive because the dominant component of the oscillating signal in the two are in fact similar. The frequency-domain method can be used to reject unambiguously the second model as a fit to the data based on the finer qualities of the oscillatory signal. In so doing, it also provides more information about why a fit is rejected and hints at how a model can be improved to better fit the data.

Another important aspect of Fourier spectra is that they can aid in determining the optimal complexity of a model structure and thereby avoid overfitting. In the examples, the spectrum of measured signal reveals many contributions from frequencies other than multiples of the base frequency. These small contributions are due to noise and do not directly carry information about the true oscillatory form. In the Fourier decomposition, they appear clearly separated from the components of the signal. Both models considered above can be seen to match only the dominant peaks of the data spectrum while ignoring the noise and can thus be argued to be reasonable attempts at modeling the data. Since the first model matches all the dominant peaks, it can in fact be argued to be an optimal description of the data despite the fact that its cost value D is close to σ. If it were made more complex by including frequency components other than those shown in Eq. S6, the result would be a model that may give a lower cost-function value but that would suffer from overfitting.

It should be stressed that the information content of an oscillating signal in its time-domain and frequency-domain representation are equivalent. It is therefore in principle possible to improve upon the time-domain analysis described above, perhaps taking into account noise levels or defining multiple cost functions capturing different aspects of signal, and thereby also conclusively rule out the simpler model as a description of the data. But the example above demonstrates that the spectrum is an appropriate tool to

carry out such model comparison analysis because it reduces model selection from a problem of comparing functions in the time domain to one of comparing a small number of peak heights, which are scalars, in the frequency domain. Model comparison can likewise be formulated in terms of ratios of peak heights, facilitating the description of an oscillating signal as compared with the time-domain representation.

The reliability of the spectrum improves with the volume of available data. Figure S2 shows how measurements of peak heights and ratios of peak heights scale with the number of data points collected. The measurements are assumed to be made in the setting described above, i.e. from a synthetic signal computed from Eq. S6 with gaussian noise, and the error bars denote the standard deviation as estimated from measurements from 1000 iterations. Points with $N = 60$ correspond to measurements analogous to those of Figure S1, i.e. for data consisting of four cycles and a resolution of 15 points per cycle. The sizes of the error bars are seen to decrease both as the number of observed cycles and as the resolution become large. They also decrease if the noise level in the signal is reduced (data not shown).

The plots show a mismatch between the measured harmonic component strengths and those of the original waveform. For example, the measured values of the second peak, shown in Figure S2A, do not converge to 12 as expected from Eq. S6. This is because there is a non-normal element in the noise which sets several of the points in the data to zero to avoid negative values. It serves to illustrate the effect spurious points can have on the estimation of the harmonic components.

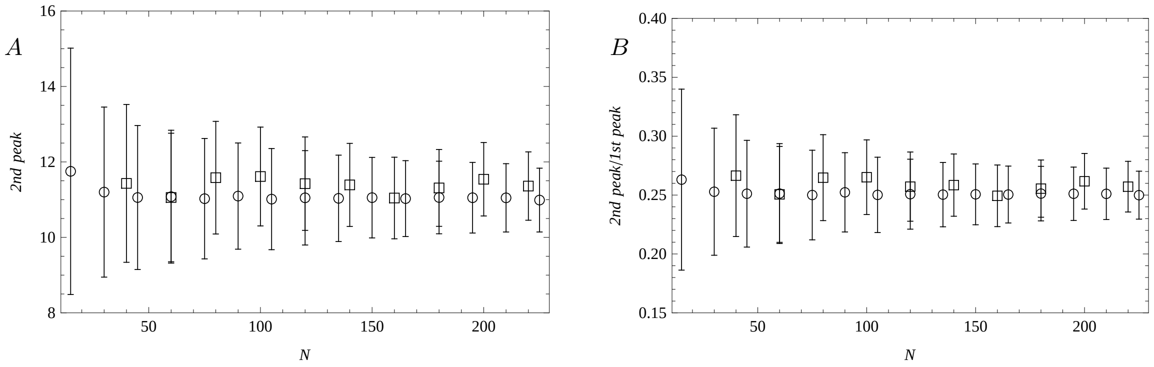

Figure S2: The reliability of information extractable from the Fourier spectrum (peak heights $H(p)$, left, and ratios of peak heights, right) increases with the number of data points points N. Circles and squares denote results obtained by observing, respectively, a varying number of cycles at fixed resolution of 15 observations per cycle and by varying the resolution at fixed observation time of four cycles.

In summary, the example shows how Fourier spectra can be applied for analyzing oscillating signals and model selection. The spectra are useful not because they contain more information than the time-domain representation, but because the information is represented in a way which is well-suited for model invalidation. The technique is applicable to all oscillating signals, but is especially suited for long time series because it is capable of describing them in terms of a relatively small number of features. In so doing, it also offers a practical way to compare signals predicted by theoretical models.
